# Wonders of the Sea

**Published:** 1885-12

**Authors:** 


### WONDERS OF THE SEA.

The sea occupies three-fifths of the surface of the earth. At the depth of about 3,500 feet waves are not felt. The temperature is the same, varying only a trifle from the ice of the pole to the burning sun of the equator. A mile down, the water has a pressure of over a ton to the square inch. If a box six feet deep were filled with sea water and allowed to evaporate under the sun, there would be two inches of salt le t on the bottom. Taking the average depth of the ocean to be three miles, there would be a layer of pure salt 230 feet thick on the bed of the Atlantic. The water is colder at the bottom than at the surface. In the many bays on the coast of Norway the water often freezes at the bottom before it does above.

Waves are very deceptive. To look at them in a storm one would think the water travelled. The water stays in the same place, but the motion goes on. Sometimes in storms these waves are forty feet high, and travel fifty miles, an hour, more than twice as fast as the swiftest steamer. The distance from valley to valley is generally fifteen times the height, hence a wave five feet high will extend over seventy-five feet of water. The force of the sea dashing on Bell Rock is said to be seventeen tons for each square yard. Evaporation is a wonderful power in drawing the water from the sea. Every year a layer of the entire sea, fourteen feet is taken up into the clouds. The winds bear their burden into the land, and the water comes down in rain upon the fields, to flow back at last through rivers. The depth of the sea presents an interesting problem. If the Atlantic were lowered 6,564 feet, the distance from shore to shore would be half as great, or 1,500 miles. If lowered a little more than three miles, say 19,680 feet, there would be a road of dry land from Newfoundland to Ireland. This is the plain on which the great Atlantic cables were laid. The Mediterranean is comparatively shallow. A drying up of 660 feet would leave three different seas, and Africa would be joined with Italy. The British Channel is more like a pond, which accounts for its choppy waves.

It has been found difficult to get correct soundings of the Atlantic. A midshipman of the navy overcame the difficulty, and shot weighing thirty pounds carries down the line. A hole is bored through the sinker, through which a rod of iron is passed moving easily back and forth. In the end of the bar a cup is dug out, and the inside coated with lard. The bar is made fast to the line, and a sling holds the shot on. When the bar which extends below the ball, touches the earth, the sling unhooks and the shot slide off. The lard in the end of the bar holds some of the sand, or whatever may be on the bottom, and a drop shuts over the cup to keep the water from washing the sand out. When the ground is reached a shock is felt as if an electric current had passed through the line.—*Electrical Review*.

---

HE has no right to think that he can enter hopefully on life who is not full of reverence before his own humanity, who does not deeply feel its wondrousness.

